# Genome sequencing: a systematic review of health economic evidence

**DOI:** 10.1186/2191-1991-3-29

**Published:** 2013-12-12

**Authors:** Martin Frank, Anne Prenzler, Roland Eils, J-Matthias Graf von der Schulenburg

**Affiliations:** 1 Leibniz University Hannover, Center for Health Economics Research Hannover, Königsworther Platz 1, 30167, Hannover, Germany; 2 German Cancer Research Center, Heidelberg, Germany

**Keywords:** Genome, Sequencing, Health economics, Cost analysis

## Abstract

Recently the sequencing of the human genome has become a major biological and clinical research field. However, the public health impact of this new technology with focus on the financial effect is not yet to be foreseen. To provide an overview of the current health economic evidence for genome sequencing, we conducted a thorough systematic review of the literature from 17 databases. In addition, we conducted a hand search. Starting with 5 520 records we ultimately included five full-text publications and one internet source, all focused on cost calculations. The results were very heterogeneous and, therefore, difficult to compare. Furthermore, because the methodology of the publications was quite poor, the reliability and validity of the results were questionable. The real costs for the whole sequencing workflow, including data management and analysis, remain unknown. Overall, our review indicates that the current health economic evidence for genome sequencing is quite poor. Therefore, we listed aspects that needed to be considered when conducting health economic analyses of genome sequencing. Thereby, specifics regarding the overall aim, technology, population, indication, comparator, alternatives after sequencing, outcomes, probabilities, and costs with respect to genome sequencing are discussed. For further research, at the outset, a comprehensive cost calculation of genome sequencing is needed, because all further health economic studies rely on valid cost data. The results will serve as an input parameter for budget-impact analyses or cost-effectiveness analyses.

## Introduction

Since the publication of a draft sequence of the human genome in 2001 [1,2] and the completion of a full sequence through the Human Genome Project in 2003 [3], the sequencing of the human genome has become a major biological and clinical research field. These research activities aim to thoroughly understand genetic diseases, e.g. cancer or rare diseases, and correspondingly to develop targeted medicines that prevent or cure these illnesses.

The literature distinguishes between several types of genome sequencing based on the extent of the analysis. The sequencing of every single base in the human genome, whole-genome sequencing (WGS), is the most comprehensive type.

Whole-exome sequencing (WES) focuses on the protein-coding portion of the genome. The exome represents only about 1% of the full genome, or approximately 30 megabases (Mb) [4]. The entire human genome consists of three billion bases. Finally single genes or specific subsets of genes can be explored. In general, the advantage of targeted sequencing approaches is the increased sequence coverage of regions of interest and a higher throughput in comparison with comprehensive sequencing methods [5,6].

Several sequencing technologies have been developed throughout the years. Since the introduction of the Sanger method [7] (first-generation sequencing) in the 1970s, these technologies have undergone various modifications. The major aims of the development of new technologies (next-generation sequencing), with regard to WGS, were to reduce the time and costs for sequencing. The Illumina HiSeq is currently, beside other sequencing platforms, widely-used for WGS.

The rapid development of these technologies and the increasing number of publications show that many scientists are involved in this research field. Competition is promoted by scientific vision and by scientific prizes offered by institutes and foundations. For instance, the often cited ‘race to the $1 000 genome’ [8,9] has played an important role. The origin of this figure lies in the vision offered by Collins et al. on behalf of the Human Genome Research Institute, formulated in 2003 [3]. The authors want to ‘provoke creative dreaming’. One vision is the ‘ability to sequence deoxyribonucleic acid (DNA) at costs that are lower by four to five orders of magnitude than the current cost, allowing a human genome to be sequenced for $1 000 or less’. In addition to the race to the $1 000 genome, the X Prize Foundation in the US wants to grant $10 million to the first team, being able to design a rapid, accurate, and economic system. The technology must be capable of sequencing 100 human genomes in ten days. Furthermore, the error rate needs to be <1 in 100 000 bases, the coverage at least 98%, and the cost no more than $10 000 for each sequenced genome [10]. However, the competition was cancelled in 2013.

The costs or, in, general the economic effect of these new technologies are of special interest especially from a public health point of view. Since there are on-going discussions on the application of these technologies to every new-born, to all newly diagnosed cancer patients or to even the broad population, it is necessary to focus on health economic aspects first in order to gain insight into the potential public health impact.

In this review, we analyse the current health economic evidence with respect to the sequencing of the human genome. Health economists distinguish between several methodological approaches. On one hand, some studies calculate the costs of new technologies and their economic burden. On the other hand, full economic evaluations go beyond pure effectiveness or cost measurements by combining assessments of costs and the consequences/outcomes of defined diagnostic procedures or interventions. Thereby, three evaluation approaches can be distinguished: cost-effectiveness analysis, cost-utility analysis, and cost-benefit analysis [11]. Cost-effectiveness analyses evaluate alternative technologies (e.g. genome sequencing versus standard diagnostic techniques) in comparing costs and a common effectiveness parameter (e.g. life-years gained through the diagnostic link of patient subgroups to specific treatments). Cost-utility analyses use utilities like quality-adjusted life-years (QALYs) as benefit parameters. The two main advantages of cost-utility analysis are that they adjust for quality of life and allow comparisons between indications. In cost-benefit analyses, not only the cost but also the benefit is measured in monetary units. However, because of the difficulty in expressing patient benefit in monetary terms, this approach is rarely used in practice. The incremental approach is a common factor in all economic evaluations: they divide the additional costs of alternative A versus alternative B by the additional benefit of alternative A versus alternative B, resulting in the incremental cost-effectiveness ratio (ICER). It states the costs per additional benefit parameter (e.g. QALYs), which need to be afforded in the case of implementing alternative A in routine care.

In the context of genome sequencing, the following health economic questions are of interest. How much do the sequencing technologies cost? Do health economic studies, which estimate the potential benefit of genome sequencing in relation to its costs, exist? What is the estimated health economic burden of these technologies? Therefore, the aim of this study was to conduct a systematic literature review of the current health economic evidence for genome sequencing. We focus not only on WGS, but also on other sequencing strategies like WES, in order to obtain a thorough overview of the current health economic evidence with respect to sequencing. Finally, based on the findings of the literature and the general knowledge on health economics, e.g. [11], we emphasize aspects that are important for health economic studies in this particular field of research.

## Materials and methods

A systematic literature search was conducted via database research of the German Institute for Medical Documentation and Information (DIMDI). DIMDI develops and operates database-supported information systems for drugs and medical devices. Hence, database research via DIMDI represents a standard for conducting international systematic literature reviews in Germany.

The following 17 German and international databases were searched: Deutsches Ärzteblatt, BIOSIS Previews, Cochrane Database of Systematic Reviews, DAHTA-Datenbank, EMBASE Alert, EMBASE, GMS, GMS Meetings, Social SciSearch, Health Technology Assessment Database, SciSearch, Krause & Pachernegg Verlagsdatenbank, MEDLINE, NHS Economic Evaluation Database, Thieme Verlagsdatenbank, and Thieme Verlagsdatenbank PrePrint.

The full-text search included publications published in German or in English from 2002 to 2012. An update was performed May 2013 in MEDLINE which listed the majority of all hits.

We used the following combination of German and English search terms, with * serving as a wildcard:

1 Title: (sequenc* OR *sequenz*)

AND

2 Text fields: (cost* OR economic* OR quality of life* OR QALY OR quality adjusted life years OR kosten* OR *ökonomi* OR lebensqualität)

Full-text publications written in English or in German were included. After the elimination of duplicates, two researchers evaluated the title and abstract of the remaining publications independently and identified possible relevant articles with health economic content. We focused on identifying cost analyses or studies that estimate the potential benefit of genome sequencing in relation to its costs (e.g. cost-effectiveness studies). The two researchers’ results were combined and disparities were discussed. After ordering the publication, we went through the full texts thoroughly.

## Results

**Figure 1:**
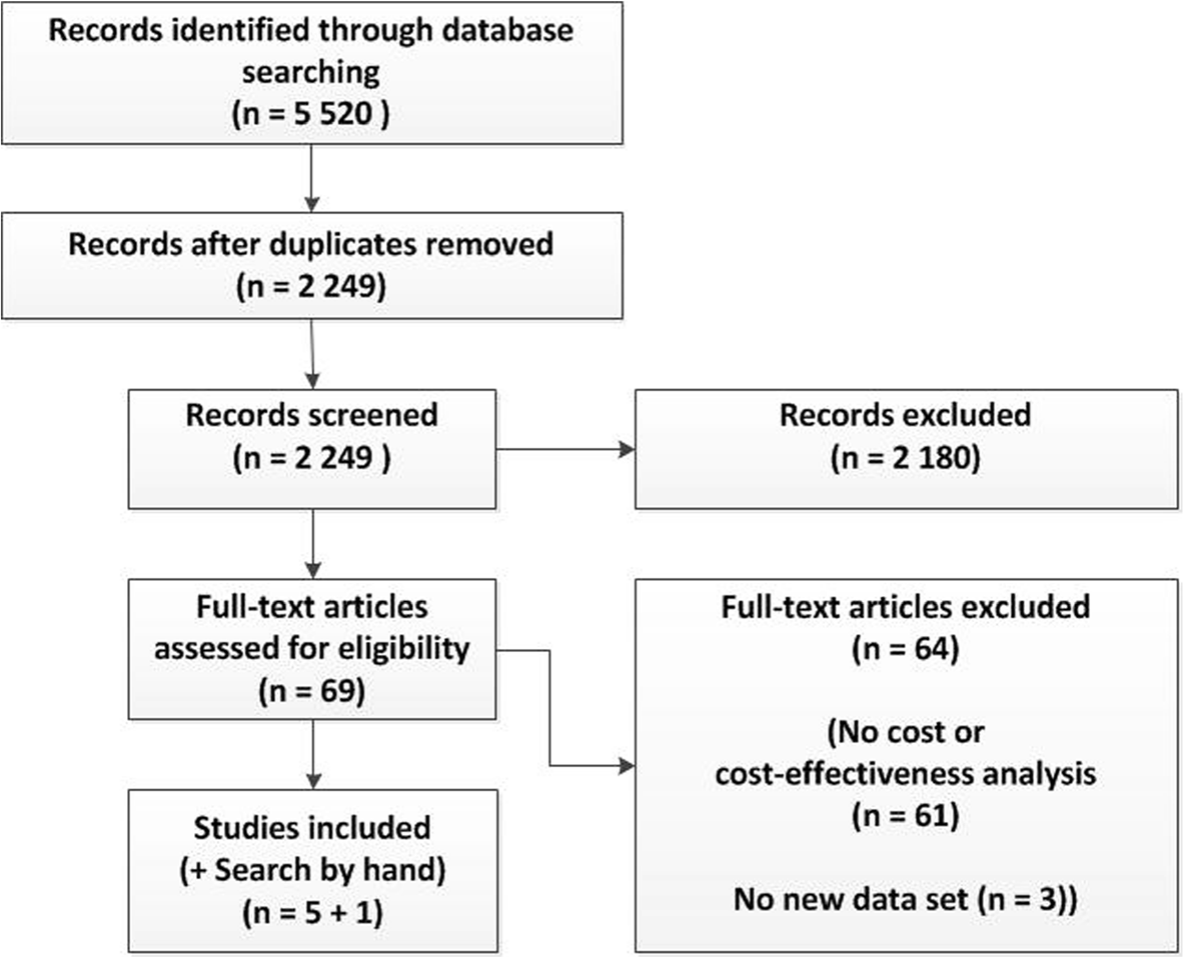
Flow diagram of articles identified and evaluated on the basis of the inclusion criteria.

The database search identified 5 520 records, of which 3 271 were duplicates. After screening the titles and/or abstracts, 2 180 publications were excluded and 69 full-text articles were assessed for eligibility. The inclusion criteria were fulfilled by five articles, which were used for this review. In addition, we included one publication from an online source. Even though it is not a full-print publication it is often cited in this context. Therefore, it was included. Figure 1 summarizes the search process.

The other full-text publications were not included because they did not contain comprehensive cost or cost-effectiveness data. Studies based on identical data were excluded from this review.

First, we focused on the five full-text publications. All five studies (see Table 1) compared cost data for the use of different sequencing platforms. No publication giving results of a cost-effectiveness analysis or a comparable study was found.

**Table 1:** Included studies

**Author**	**Title**	**Bibliography**
Glenn TC	Field guide to next-generation DNA sequencers	Mol Ecol Resour 2011; 11: 759-769
Kircher M, Kelso J	High-throughput DNA sequencing—concepts and limitations	Bioessays 2010; 32: 524-536
Pareek CS, Smoczynski R, Tretyn A	Sequencing technologies and genome sequencing	J Appl Genetics 2011; 52: 413-435
Shendure J, Ji H	Next-generation DNA sequencing	Nat Biotechnol 2008; 26: 1135-1145
Tucker T, Marra M, Friedman JM	Massively Parallel Sequencing: The Next Big Thing in Genetic Medicine	Am J Hum Genet 2009; 85: 142-154

One major distinction between the reviewed studies is the consideration of acquisition costs. Tucker et al. [12] state that commercially available instruments cost between $400 000 and $1 350 000 each, not including costs associated with the software, training, and data transfer as well as storage. However, Glenn [13] states that the costs for the second-generation platforms currently range from $ 49 500 to $ 695 000. Moreover, the cost calculation process differs between the included studies. The cost calculation process was not described in a transparent way in any of the reviewed studies.

Costs per Mb for the application of the sequencing platforms differ. Moreover, the cost per Mb for the use of identical sequencing technologies varies between the identified studies, because they might include different factors like depreciation costs, personal costs, or lab facility costs. Using the example of the Roche/454 GS FLX Titanium, Glenn determined the cost per Mb of $12.40. In contrast, Pareek et al. [14] calculated a cost per Mb of $84.39. The Sanger method is the most expensive sequencing technology, costing approximately $500 per Mb [18]. The human genome consists of approximately 3 000 Mb. Hence, the WGS process would cost approximately $1 500 000. However, genome sequencing with the Illumina HiSeq 2000 platform seems to be one of the least expensive alternatives, costing $0.10 per Mb [13]. In this case, only $300 are required for the WGS process. Technical specifications differ between the sequencing platforms. Especially raw accuracy is important for the sequencing quality. Moreover, a high throughput per day is necessary for WGS. In Table 2, we merged the results of the included studies.

**Table 2:** Results of the reviewed studies

	**Author (publication year)**	**Throughput Mb/day**	**Bases/read**	**Raw accuracy**	**Cost per instrument**	**Cost per Mb approximately**
Sanger	Kircher et al. (2010)	6	800	0.99991	-	$500.00
Roche/454 GS FLX Titanium	Shendure et al. (2008)	-	250	^-^	$500 000	$60.00
	Tucker et al. (2009)	1 200	400	0.99	-	$84.40
	Kircher et al. (2010)	750	400	0.9991	-	$20.00
	Glenn (2011)	-	400	0.99	-	$12.40
	Pareek et al. (2011)	1 000	400	0.995	$500 000	$84.39
Illumina Genome Analyzer II/IIx	Tucker et al. (2009)	2 500	75+	0.985	-	$6.00
	Kircher et al. (2010)	5 000	100	0.991	-	$0.50
Illumina Solexa	Shendure et al. (2008)	-	36	-	$430 000	$2.00
	Pareek et al. (2011)	1 500	36	0.985	$400 000	$5.97
Illumina HiSeq 2000	Glenn (2011)	-	100 + 100	0.99	-	$0.10
Applied Biosystems SOLiD 4	Shendure et al. (2008)	-	35	-	$591 000	$2.00
	Pareek et al. (2011)	1 850	35	0.9994	$525 000	$5.81
	Tucker et al. (2009)	2 500	50	0.994	-	$5.80
	Kircher et al. (2010)	5 000	50	0.991	-	$0.50
Life Technologies SOLiD 5500	Glenn (2011)	-	75 + 35	0.99	-	<$0.08
Life Technologies SOLiD 5500xl	Glenn (2011)	-	75 + 35	0.99	-	<$0.07
Helicos HeliScope	Shendure et al. (2008)	-	30	-	$1 350 000	$1.00
	Tucker et al. (2009)	3 100	30-35	0.99	-	-
	Kircher et al. (2010)	5 000	32	0.99	-	$0.50
	Glenn (2011)	-	35	-	-	-
	Pareek et al. (2011)	2 500	>1000	>0.99	-	-
Polonator	Shendure et al. (2008)	-	13	-	$155 000	$1.00

Finally, the internet source, that we have already mentioned, was evaluated. The National Human Genome Research Institutes webpage informs about longitudinal sequencing costs and updates the data on a regular basis [15]. Two cost categories are distinguished and defined as production and non-production costs. The cost for sequencing decreases faster than Moore’s law [16], which was $0.06 per Mb and $5 826 per genome in April 2013. However, the cost calculation process is not stated, and no cost values are given for the separated categories.

The results varied between assessment dates due to the decreasing costs of sequencing technologies. Technical adjustments to the sequencing platforms, such as coverage, differ between the included studies as well. Although some authors have gathered cost data on the existing sequencing platforms, the real costs for the whole sequencing workflow remain a ‘black box’. And the reason for this is, that the numerous resources connected to genome sequencing are insufficiently incorporated into the cost calculations. To conduct an adequate cost analysis, all necessary resources required for the application of WGS and their specific values should be included. However, inclusion of resources for the cost analysis depends on the perspective of the evaluation. We will stress this aspect later in the discussion.

There are no accepted standards for what kind of measures the companies need to report [13]; therefore, it is difficult to compare the technologies from different providers. In addition, Shendure et al. [17] state that a simple comparison between ‘per base’ costs may be misleading, because statistically more accurate bases may be worth more than less accurate bases. Furthermore, a direct comparison of error rates is problematic, as most companies report errors based on sequence reads of the particular templates that are favourable for their platform. They use these particular templates for quality control [13]. However, for health economic evaluations, it is important to include valid error rates: these are comparable to the sensitivity and specificity of diagnostic instruments, which play an important role in evaluations. Hence, Glenn [13] suggested that it would be useful to develop a standard set of conditions, analyses, and templates to compare next-generation-platforms, chemistry, and software upgrades.

The substantial–but numerically unknown–expenditures in data management and analysis, time, and personnel, which are not yet included in the analysis, also contribute to the ‘black box’. As Glenn [13] shows, computational resources required to process and analyse data vary between the platforms because short reads require analysis that is more intensive. For larger amounts of data, it is even necessary to use high-performance computing clusters (HPCCs). Furthermore, costs for the instrument acquisition and maintenance need to be considered, depending on the perspective of the analysis. According to Kircher et al. [18], these factors remain important challenges; hence, inclusion of financial investments needs to be discussed, depending on the study perspective. Glenn’s figures [13] also assume that the equipment will be housed in well and properly functioning laboratory. If genome sequencing is to be established in a new facility, then the costs will increase considerably. In this context, Tucker et al. [12] emphasise that the initial costs, which are needed to set up a massively parallel sequencing platform, are extremely high.

Mardis [19] highlightes challenges regarding the high effort in analysing sequenced data. Thereby it is important to distinguish between the computational analysis in order to identify important gen variants and the manual analysis to interpret the data. Mardis emphasizes that for the both analyses many professionals are required, including molecular and computational biologists, geneticists, pathologists, and physicians with extensive knowledge of the disease and treatment modalities. Moreover, these professionals are typically supported by research nurses, genetic counsellors, and information technology (IT) and systems support specialists. Furthermore, Mardis claimes that aspects of data analysis, including the filtering of reliable variants and their clinical validity as well as the communication of results to the treating physician and ultimately to the patient, are underestimated problems and require many resources. Hence, the costs for the sequencing process itself seem to be only a marginal part of the whole cost of the WGS process. This point is also emphasized by Stone and Levenson [20]. They say that genomic data have no practical use without a complicated, costly bioinformatics analysis by specialists.

Another important factor is storage costs. The storage of one person’s WGS data requires approximately 2.5 terabytes of disk space. Computer systems to analyse and store data are costly [21]. Glenn [13] assumes that the storage costs may even be higher than the analysis costs. If the cost of the sequencing processes decreases and the quality of sequence data and analysis improves, re-sequencing the patient rather than re-evaluating the old data may be more appropriate [21]. However, if storage facilities improve in the future, the storage and re-analysis of data might become more efficient. In many situations, sample availability restricts or prohibits re-sequencing. In this context, it is important to stress the highly dynamic process of the technologies and the difficulties in forecasting further developments. For example, Carr et al. [22] outline the technical progress of length and costs of base pairs over the years. Wetterstrand [15] also documentes the development. However, future developments are difficult to predict. Nevertheless, data analysis and storage should be included in a comprehensive cost analysis.

Bick and Dimmock [21] also raise an issue regarding the confirmation of the results. A high possibility of false-positive results leads to insecurity and it has a tremendous impact on health care costs. If other, more established methods are needed to verify the results of WGS, one must include the costs of these methods in a health economic evaluation. For example, when analysing a recessive disorder that is a compound heterozygote, the two putative pathogenic variants will need to be confirmed. Furthermore, Brick et al. state that the cost of custom sequencing for each mutation is about $ 200. The expectation is that, as the technology matures, the need for confirmatory testing will diminish. However, this is not the case yet.

Other authors focus on challenges in the clinical application of WGS and emphasize the difficulties in integrating the patient into the information system [19,22]. Current clinical tests, e.g. an human immunodeficiency virus (HIV)-test, provide patients with meaningful information about a particular disorder; however, the results of WGS cannot easily be translated into meaningful information yet. With respect to new-born screening, Valle [23] is concerned whether large-scale genomic sequencing provides any information with clinical use in infants. Furthermore, patients weigh individual risks differently; they may have difficulties in understanding and applying probabilities and risks. Finally, every person has some kind of genetic predisposition to some kind of disorder; this knowledge could result in several social problems, involving reproductive decisions, insurance contracts, or employment, example.g. Another issue in clinical practice will be shortcomings of qualified manpower in the whole sequencing process. The need for professionals with different qualifications is discussed above. Hence, the question is who is able to provide accurate information to patients before sequencing and a skilled interpretation of the results afterwards. It seems that there are several natural barriers to the expansion of gene sequencing technologies into clinical practice.

## Discussion

Based on the literature findings as well as the general knowledge on health economics e.g. [11], we developed a list of aspects that needs to be considered before conducting a health economic analysis of genome sequencing. As stated in the introduction, some health economic studies consider only cost parameters when calculating costs of new technologies and their economic burden. However, full economic evaluations go beyond pure cost measurements by combining costs and the consequences/outcomes of defined diagnostic procedures or interventions. In the future, this list will help researchers who plan to conduct a health economic analysis.

### Overall aim: improvement of clinical research or routine care?

Currently, genome sequencing is mainly conducted for clinical research proposes. However, in general, health economic evaluations are made for clinical practice. They are necessary to decide whether the additional benefit of a new diagnostic technology is worth the additional costs and hence eligible for reimbursement in routine care. This separation between research and routine care is crucial. For instance, in Germany the Statutory Health Insurance, which decides on the reimbursement of new diagnostic and therapeutic technologies, is not allowed by law to support research. It is only in charge of the health care of the insurants. Hence, if (whole) genome sequencing for routine care was to be reimbursed, it would be important to emphasise the direct benefit of the technology for patients and the comprehensive costs of its application for health care systems. The following aspects all refer to the overall aim of receiving reimbursement for genome sequencing in clinical practice.

### Technology

As the previous findings show, different technologies for WGS (Roche/454, Helicos, etc.) vary considerably. Hence, within the health economic evaluation it is very important to clearly state which technology is used and to clarify technical calibrations like coverage.

### Population/indication

A famous concept, developed for epidemiologic and clinical studies, is the so-called PICO-framework. PICO stands for Population Indication Comparator Outcomes and it is an important tool in evidence-based medicine. This framework is also helpful for health economic studies. It is important to focus on a specific population with a definite indication. In general, diagnostic or therapeutic technologies are not reimbursed for every kind of population and indication. Comparable to clinical approval by the European Medicines Agency or the U.S. Food and Drug Administration, reimbursement approval is very restricted. This emphasises the importance of clearly describing the populations and indications that benefit from the new technology.

### Comparator

Comparable to a classical randomized controlled study, a new technology should always be compared to another existing technology. This is important to identify additional benefits and additional costs of the new technology. It is advisable to take as a comparator a technology that is already reimbursed by health insurance. Another possibility for a comparator is ‘do nothing’, if no eligible standard care for comparison is available. Hence, for genome sequencing, a reasonable comparator could be ‘not to conduct a diagnostic test’.

### Alternatives after sequencing

In this section, we want to emphasize the importance of obtaining actionable results from the sequencing process. Depending on the sequencing result, the diagnostic procedure must result in a preventive or therapeutic action. If, independent of the result, the output is only ‘nice to know’, then, the use of the diagnostic procedure may not be justified. There may be an exception if a patient has ailments for some time and their cause is unclear. Although the diagnosis may not lead to therapeutic actions due to the incurable nature of the disease (e.g. Huntington's disease), future diagnostic procedures could be avoided and the patients would know the cause of their indisposition. This may positively affect their health-related quality of life.

Another important aspect is that the alternatives after sequencing mainly depend on the so-called positive list for incidental findings. This list includes all the diagnoses that should be communicated to the physicians/patients after sequencing. Hansson [24] recommends investigations into whether evidence-based prevention or treatment is available for the particular gene finding. Otherwise, a clinical utility is questionable. Hansson also states that DNA donors should be informed that incidental findings must first be confirmed and translated into clinical application before information is communicated to them. Nevertheless, the more comprehensive the list of communicable incidental findings is, the more complex the health economic evaluation will be, because various treatment options, depending on the different diagnoses, including their effectiveness, need to be considered. However, the discussion of which criteria should determine the indication list is continuing [25,26].

### Outcomes

One can distinguish between surrogate-parameters and patient-relevant outcomes. Surrogate-parameters are measureable patient characteristics intended to substitute for a patient-relevant clinical endpoint such as median overall-survival. An example of a surrogate-parameter in the context of genome sequencing is the number of detected gene mutations. However, the role of patient-relevant outcomes is increasing. Patient-relevant outcomes capture those outcomes that are relevant to the patient, such as health-related quality of life or life expectancy. In the case of WGS, the identification of the patient-relevant outcome is not trivial. For instance, an earlier diagnosis does not necessarily lead to better health-related quality of life or higher survival. Furthermore, the patient benefit that is generated depends largely on the subsequent treatment options. Importantly, findings that are interesting for research purposes and that may lead to patient benefits in the future are not to be included in such a health economic evaluation.

### Probabilities

On the basis of the previous aspects, it is important to identify reliable and valid probabilities. Based on the sequencing data generated, how much higher is the risk that a person will develop cancer or other diseases? How valid and reliable is this analysis? Because sequencing technologies also produce errors, the resulting false-positive results and their clinical and economic impact need to be considered in a health economic analysis. In addition, even if a variant is accurately defined, further evidence of both clinical validity and utility are needed if its discovery is to be meaningful. This aspect emphasizes the immense need for research on valid clinical data. Without these data, a full economic evaluation is not feasible.

### Costs

Finally, cost data are an obligatory part of health economic evaluations. As discussed before, no valid cost analysis exists so far.

A cost analysis is subdivided into the identification, quantification, and valuation of resources. In this context, it is important to stress that the number of resource types depend on the study perspective. When choosing the perspective of social health insurance, the reimbursement scheme is of interest. From a management point of view, a full cost calculation needs to consider not only the pure sequencing costs, but also the resources required for acquisition, maintenance, and analysis. Because reimbursement of whole-genome sequencing by social health insurance is not yet foreseen, the identification and valuation of all the resource connected to sequencing might be the first step. This calculation could then serve as the basis for subsequent reimbursement.

Such a calculation implies that, first, the whole sequencing process needs to be determined and documented; hence, the resources must be quantified and valued. Furthermore, all of the steps in the cost analysis need to be documented separately to conduct a transparent investigation. As the valuation mainly depends on the respective nation, it is possible to transfer the results to another country’s specific health care settings by simply adjusting the valuation of the resources. In addition, the cost model must allow different kinds of sequencing platforms and sensitivity analyses. In sensitivity analyses, uncertain variables are varied in order to check the robustness of the calculation and to identify the cost-driving variables. Hereby, it is expected that the high throughput sequencing of gene panels will be less costly than WGS.

As all further health economic studies rely on valid cost data, at first, a detailed cost study is needed, which incorporates all actions connected to genome sequencing–starting from informed consent and sampling to the point of communicating the results. The results could also serve as an input for a budget-impact analysis.

Conducting a valid cost analysis is only the first step. Full health economic evaluations must also include the follow-up costs as well as the benefits for patients. However, before that, considerable research is needed to improve the clinical data, which are an important input for health economic studies.

Because of the comprehensive and complex health economic challenges connected to genome analyses, health economic research will and must grow in the future to explore the benefits and costs of these novel technologies for patients and society.

## Conclusion

This review indicates that it is still a long way to achieve the $1 000 genome–when all costs are considered, we hypothesise that this price will not be achieved. Nevertheless, even if the $1 000 genome was realizable one day, e.g. in the German context, it would cost around $ 660 million to sequence every new-born and $ 500 million to sequence every new cancer patient [27], which would have a major public health impact. Hence, there is further research necessary to justify these costs in relation to the additional benefit for the patients.

## Compliance with ethics guidelines

Since we conducted a systematic literature review and no experiment, there exists no conflict with ethics guidelines.

## Abbreviations

DIMDI: German institute for medical documentation and information; DNA: Deoxyribonucleic acid; HIV: Human immunodeficiency virus; HPCC: High-performance computing cluster; ICER: Incremental cost-effectiveness ratio; IT: Information technology; Mb: Megabase; QALY: Quality-adjusted life-year; WES: Whole-exome sequencing; WGS: Whole-genome sequencing.

## Competing interests

The authors declare that they have no competing interests.

## Authors’ contributions

MF und AP: literature search and analysis, discussion, writing of the manuscript. RE and JMS: discussion, critical review. All authors read and approved the final manuscript.
